# Ultrastructural Changes during the Life Cycle of *Mycoplasma salivarium* in Oral Biopsies from Patients with Oral Leukoplakia

**DOI:** 10.3389/fcimb.2017.00403

**Published:** 2017-09-21

**Authors:** Harumi Mizuki, Ryosuke Abe, Toshinari Mikami

**Affiliations:** ^1^Division of Oral and Maxillofacial Surgery, Department of Oral and Maxillofacial Reconstructive Surgery, School of Dentistry, Iwate Medical University Morioka, Japan; ^2^Division of Anatomical and Cellular Pathology, Department of Pathology, Iwate Medical University Shiwagun, Japan

**Keywords:** *Mycoplasma*, *Mycoplasma salivarium*, oral leukoplakia, electron microscopy, immunoelectron microscopy, infection, oral mucosa, life cycle

## Abstract

Bacteria in genus *Mycoplasma* spp. are the smallest and simplest form of freely replicating bacteria, with 16 species known to infect humans. In the mouth, *M. salivarium* is the most frequently identified species. *Mycoplasma* spp. are parasites with small genomes. Although most of the *Mycoplasma* spp. that infect humans remain attached to the host cell surface throughout their life cycle, we have previously reported the presence of *Mycoplasma salivarium* in the epithelial cells of oral leukoplakia and oral lichen planus. However, the mechanism underlying the pathogenicity of *M. salivarium* has remained unclear. Further studies are needed to identify the process of infection of human cells and the stages in the life cycle of *M. salivarium*. Electron microscopy (EM) is the method of choice for morphological investigation of *Mycoplasma* spp. in cells or tissues. This study was performed to clarify and detail the ultrastructure of *M. salivarium* in tissue biopsies of oral mucosal leukoplakia, using three EM methods: (1) a standard EM processing method; (2) an ultracryotomy and immunolabeling method; and (3) the LR White resin post-embedding and immunolabeling method. This study included five oral leukoplakia tissue samples showing hyperplasia and hyperkeratosis. Although there was some variation in ultrastructural appearances between the three EM methods used, there were four ultrastructural appearances that are believed to reflect the stages of the *M. salivarium* life cycle in the epithelial cells of the oral mucosa: (1) small, electron-dense cellular-like structures or elementary bodies of *M. salivarium*; (2) large structures of *M. salivarium*; (3) *M. salivarium* organisms in cell division; (4) the sequence of events in the life cycle of *M. salivarium* that includes: (a) elementary bodies of *M. salivarium* deep in the oral mucosal epithelium; (b) replication by binary fission and daughter cell division from the elementary bodies; (c) maturation or degeneration of *M. salivarium* in the epithelial cells mainly in the upper part of the epithelium; and (d) death of the organisms in the granular and/or keratinized layer. These ultrastructural images may provide a useful reference for the identification of *M. salivarium* in diagnostic cytology or biopsy material.

## Introduction

Bacteria in the genus *Mycoplasma* spp. are the smallest and simplest form of freely replicating bacteria and are characterized by the lack of a rigid cell wall, being bound by a single plasma membrane (McElhaney, 1992). There are 16 species of *Mycoplasma* known to infect humans, including *M. pneumoniae, M. salivarium, M. buccale, M. orale, M. faucium, M. lipophilum*, and *M. fermentans*, which are also commensal organisms of the oropharynx (Blancherd and Bebear, 2002). In the mouth, *M. salivarium* and *M. orale* have been identified more frequently than the other species of *Mycoplasma* (Watanabe et al., 1986).

*Mycoplasma* spp. have extremely small genomes that range in size from 580 to 2,200 kb, with their metabolic options for replication and survival being limited by their small genome (Rottem, 2003). *Mycoplasma* spp. exhibit strict host and tissue specificities. Most of the organisms that infect humans remain attached to the host cell surface throughout their life cycle, except for *M. pneumoniae, M. penetrans, M. genitalium, M. hominis*, and *M. fermentans*, which may reside within non-phagocytic cells under certain circumstances (Razin et al., 1998).

Recently, we have demonstrated the localization of *M. salivarium* in the epithelial cells of oral leukoplakia tissue using immunohistochemistry (IHC) and immunoelectron microscopy (IEM) (Mizuki et al., 2015). Oral leukoplakia is the most common pre-malignant disorder, or potentially malignant disorder, of the oral mucosa (Mortazavi et al., 2014). Currently, oral leukoplakia is defined as “a white plaque of questionable risk having excluded (other) known diseases or disorders that carry no increased risk for cancer” (Warnakulasuriya et al., 2007). The histopathology of oral leukoplakia, following oral mucosal biopsy, shows atrophy or hyperplasia that may or may not include epithelial dysplasia (Warnakulasuriya et al., 2007). Therefore, oral leukoplakia is a clinical diagnosis that requires tissue biopsy and histological diagnosis to rule out a diagnosis of squamous cell carcinoma-*in-situ* or invasive squamous cell carcinoma (Warnakulasuriya et al., 2007).

The presence of *M. salivarium* in the epithelial cells of oral leukoplakia is also associated with hyperkeratosis and hyperplasia of the oral mucosal epithelium, but the role of *M. salivarium* in the development of these oral mucosal epithelial changes has been poorly understood (Mizuki et al., 2015). It has also been previously assumed that *M. salivarium* has no pathogenicity (Blancherd and Bebear, 2002). However, we have recently demonstrated the localization of *M. salivarium* within the oral mucosal epithelium and in the area of subepithelial lymphocyte infiltrate in tissue biopsy material of oral lichen planus, which is another cause of oral mucosal plaques, using IHC (Mizuki et al., 2017). Although it was unclear from this recent study whether or not *M. salivarium* was within the lymphocytes or in the intercellular spaces between the lymphocytes in lichen planus, our finding of the localization of *M. salivarium* in the area subepithelial chronic inflammation suggests that it is an infectious organism capable of causing a host tissue response (Mizuki et al., 2017).

Immunohistochemistry is useful for the identification of *Mycoplasma* spp. in tissue sections (Mizuki et al., 2015). However, the results of IHC can vary according to the specificity of the primary antibodies used, the availability of the antigen to antibody localization, and the sensitivity of IHC detection methods. Although the area of the tissue sample that can be viewed is limited, electron microscopy (EM) is a reliable method for identifying *Mycoplasma* spp. in cells or tissues (Phillips, 1978).

However, there have been several published studies that have shown that standard EM tissue processing and imaging methods for *Mycoplasma* spp. grown in broth or on agar, may result in variable findings that may, in some cases, be non-specific (Domermuth et al., 1964; Anderson and Barile, 1965; Hummeler et al., 1965; Knudson and MacLeod, 1970; Nakamura and Kawaguchi, 1972; Furness et al., 1976). However, until our recently published study, there have been no *in vivo* or tissue biopsy ultrastructural studies to image *Mycoplasma* spp. in the epithelium of the oral mucosa (Mizuki et al., 2015, 2017). Currently, there is no available reference source of ultrastructural images of *Mycoplasma* spp. and the *in vivo* localization in cells or tissues.

Therefore, although several previous authors have observed oral leukoplakia tissue samples using standard EM methods, none of these previous studies have shown the presence of *Mycoplasma* spp. in oral leukoplakia tissues, and no previous studies have demonstrated the ultrastructural features of the life cycle of this organism in human tissue (Silverman, 1967; Hashimoto et al., 1968; Banoczy et al., 1980; Rodriguez et al., 1984; Jungell et al., 1987; Kannan et al., 1993; Tamgadge et al., 2012). It is possible that the ultrastructural features of *Mycoplasma* spp. in epithelial cells of oral leukoplakia tissue has been poorly investigated because of the assumption that the ultrastructural features described from growth broth or on agar were no different from tissue samples. Also, in addition to the EM images of *Mycoplasma* spp. in the epithelial cells of oral leukoplakia tissue, which are now shown to be optimally demonstrated using an ultracryotomy method for EM in our previous study, *Mycoplasma* spp. grown on agar and in broth have previously used a standard method for EM (Mizuki et al., 2015).

Immunoelectron microscopy has been developed as a technique for specific identification of antigens in tissue, including infectious micro-organisms. Currently, three main methods are used for tissue preparation for IEM: the ultracryotomy method; the pre-embedding method using epoxy resin; and the post-embedding method using LR White acryl resin (De Paul et al., 2012). The ultracryotomy method for EM with immunogold labeling that we have used to identify *M. salivarium* is a suitable method for IEM due to the improved permeability of antibodies into the cells of the tissue sections (Akagi et al., 2006). However, this method can be technically difficult and requires specialized apparatus, which explains why it is not widely used. In contrast, a more standard or conventional EM method, which has been widely used since the 1960s, comprises double fixation with paraformaldehyde/glutaraldehyde and osmium tetroxide, followed by dehydration, and embedding in epoxy resin. The pre-embedding method using the standard EM method is not always suitable for IEM due to the limited penetration of some antibodies into tissue samples (Zhong et al., 2013). For these reasons, post-embedding immunolabeling on sections prepared by fixation with paraformaldehyde and/or glutaraldehyde, dehydration, and embedding in LR White resin, is often used for IEM. Use of the LR White resin embedding gives better permeability of antibodies into the tissue section than epoxy resin embedding (Timms, 1986). Also, the LR White resin embedding method does not require specialized apparatus for thin sectioning, unlike ultracryotomy.

The aim of this study was to clarify and detail the ultrastructure of *M. salivarium* in the epithelial cells of oral mucosa using five oral leukoplakia tissue biopsy samples, where the presence of *M. salivarium* was confirmed in advance by IHC. Three EM methods were used in this study: (1) a standard EM processing method; (2) an ultracryotomy and immunolabeling method; and (3) the LR White resin post-embedding and immunolabeling method. The ultrastructural images obtained may provide a useful reference for the identification of *M. salivarium* in diagnostic cytology or biopsy material.

## Materials and methods

### Patients and oral tissue biopsies

The study protocol was approved by the Ethical Committee of the School of Dentistry at Iwate Medical University (No. 1223). Written informed consent was obtained from all the study participants.

Oral leukoplakia was identified visually as a homogeneous oral mucosal white patch in nine patients who had a diagnostic incision or excision biopsy and who agreed to participate in the study (Table 1). The patients had no systemic diseases or disorders and were treated at the Department of Oral and Maxillofacial Surgery, Iwate Medical University Hospital. The biopsies were divided into two parts; one was used for histopathological examination by light microscopy and IHC, and the other was used for EM.

**Table 1:** Clinical features of oral leukoplakia samples used in this study.

**No**	**Age (years)**	**Sex**	**Location of the lesion**	**Clinical type of lesion**	**History of the lesion**
1	63	M	Tongue	Homogeneous	New presentation
2	71	F	Cheek	Homogeneous	Long-standing lesion
3	59	F	Tongue	Homogeneous	Long-standing lesion
4	73	M	Gingiva	Homogeneous	Long-standing lesion
5	77	M	Gingiva	Homogeneous	Long-standing lesion

### Light microscopy: hematoxylin and eosin (H&E) staining and IHC

Specimens of oral biopsy tissues were fixed in formalin and embedded in paraffin wax. Tissue sections were cut at 4 μm as serial sections and mounted on MAS-coated slides (Matsunami Glass Ind., Ltd., Osaka, Japan) for H&E staining and IHC. The sections were deparaffinized, rehydrated, and then stained with H&E for histopathological examination. Five oral leukoplakia samples that exhibited hyperplasia and hyperkeratosis, without epithelial dysplasia or Candida infection, were chosen for IHC and EM. Invasive oral mucosal Candida was excluded by light microscopy of the oral biopsy material using standard H&E tissue section staining, and periodic acid-Schiff (PAS) staining for fungal organisms. The clinical features of five patients are shown in Table 1.

Before examination by EM, the localization of *Mycoplasma salivarium* in the epithelium of the biopsy tissue sections was confirmed by IHC with anti-*M. salivarium* monoclonal antibodies (MAbs) (Mab 7-6H) produced in our laboratory. IHC was performed using a biotin-free tyramide-catalyzed signal amplification (CSAII) system (Dako, Carpinteria, CA, USA), as described previously (Mizuki et al., 2015). Briefly, tissue sections were incubated with anti-*M. salivarium* monoclonal antibody (culture supernatant) without dilution. The sections were then incubated with horseradish peroxidase (HRP)-conjugated polyclonal rabbit anti-rat immunoglobulin (1:100). The sections were incubated with 3,3'-diaminobenzidine tetrahydrochloride (DAB) solution, counterstained with hematoxylin, and then observed by light microscopy. The DAB resulted in brown chromogenic localization of the primary antibody that could be visualized by light microscopy.

Five samples of normal oral mucosa with normal macroscopic appearance and normal histology were used as controls for light microscopy and IHC. These tissue samples were from the patients recruited into the study; the age range of the five patients who provided control tissue (3 men and 2 women) was between 45 and 71 years.

### Electron microscopy: the standard method

The small samples of leukoplakia tissues were immediately fixed with 2% paraformaldehyde and 2.5% glutaraldehyde in 0.1 M phosphate buffer (PB) (pH 7.4) at room temperature for 2 h. Following washing with 0.1 M PB twice for 10 min at 4°C, the specimens were post-fixed in 1% osmium tetroxide in 0.1 M PB at 4°C for 2 h. Dehydration of the specimens through a graded series of ethanol solutions was followed by substitution with N-butyl glycidyl ether (QY-1) (Nisshin EM, Tokyo, Japan) twice for 15 min and then with QY-1 and epoxy resin (Epon 812) (TAAB Laboratories, Aldermaston, Berks, UK) mixed 1:1 overnight at room temperature and final infiltration with Epon 812 for 6 h at room temperature. Polymerization was performed by incubation at 60°C for 3 days.

The resin-embedded samples were cut into ultrathin sections (70–80 nm thickness), and the sections were collected on copper grids (Nisshin EM). The sections were stained with 1% uranyl acetate in 0.1 M PB for 30 min and 1% lead nitrate in 0.1 M PB for 5 min, and they were then observed under an H-7650 electron microscope (Hitachi High-Technologies, Tokyo, Japan).

### IEM: the ultracryotomy immunolabeling method

The oral leukoplakia tissues were used for the ultracryotomy and immunolabeling method, as described previously (Mizuki et al., 2015). Briefly, the oral leukoplakia samples were fixed with 4% paraformaldehyde in 0.1 M PB (pH 7.4). The tissue samples were then immersed in 0.1 M PB containing 30% sucrose, transferred to 20% polyvinylpyrrolidone (PVP) and 1.84 M sucrose in 0.1 M PB. The tissue samples were frozen with liquid propane at −185°C and were cut into ultrathin sections. Sections were incubated with 10% normal goat serum (Santa Cruz Biotechnology, Santa Cruz, CA, USA) to block non-specific localization of primary antibody, using 0.1 M Tris-buffered saline (TBS), the sections were incubated in 0.1 M TBS containing anti-*Mycoplasma* spp. polyclonal antibody (1:1,000). The sections were incubated in 0.1 M TBS containing colloidal gold (diameter: 5 or 10 nm) which was conjugated to a secondary antibody (1:100), goat anti-rabbit IgG (BBI Solutions, Cardiff, UK). Immunogold-labeled sections were observed under H-7100 and H-7650 electron microscopes (Hitachi High-Technologies).

### IEM: the LR white resin embedding and immunolabeling method

The oral leukoplakia tissues were cut into small pieces and were fixed with 4% paraformaldehyde in 0.1 M PB (pH 7.4), at room temperature for 4 h. Following washing with 0.1 M PB twice for 10 min at 4°C, dehydration of the specimens through a graded series of ethanol solutions was performed. The substitution was with 100% ethanol, and LR White resin (London Resin Company Ltd., London, UK) mixed 1:1 at 4°C for 1 h, and finally infiltration with LR White resin at 4°C overnight. Polymerization was performed by incubation at 60°C for 24 h. The resin-embedded samples were cut into ultrathin sections (70–80 nm thickness), and the sections were collected on nickel grids (Nisshin EM).

Post-embedding immunogold labeling was performed on the sections. After blocking with 10% normal goat serum (Santa Cruz Biotechnology) in 0.1 M TBS for 2 h at room temperature, the sections were incubated in 0.1 M TBS containing anti-*Mycoplasma* spp. polyclonal antibody (1:250) at 4°C for 2 days. Following washing with 0.1 TBS four times for 10 min, the sections were incubated in 0.1 M TBS containing the colloidal gold-conjugated secondary antibody (gold particle diameter: 10 nm), goat anti-rabbit IgG (BBI Solutions) (1:100). The sections were then washed with 0.1 M TBS for 10 min, and distilled water for 10 min. The sections were stained with 1% uranyl acetate in 0.1 M PB for 30 min and 1% lead nitrate in 0.1 M PB for 5 min, and were observed under H-7650 electron microscopes (Hitachi High-Technologies).

**Figure 1:**
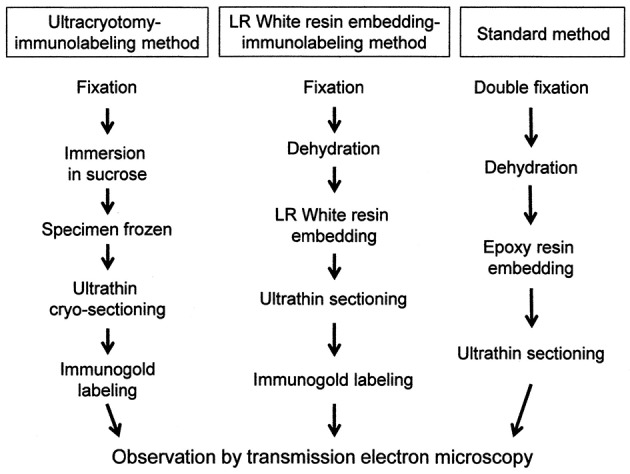
A flowchart showing the process of sample preparation for electron microscopy in each method.

The process of preparation of ultrathin sections for EM using the three methods described in this study is shown in Figure 1.

## Results

### Light microscopy and IHC

**Figure 2:**
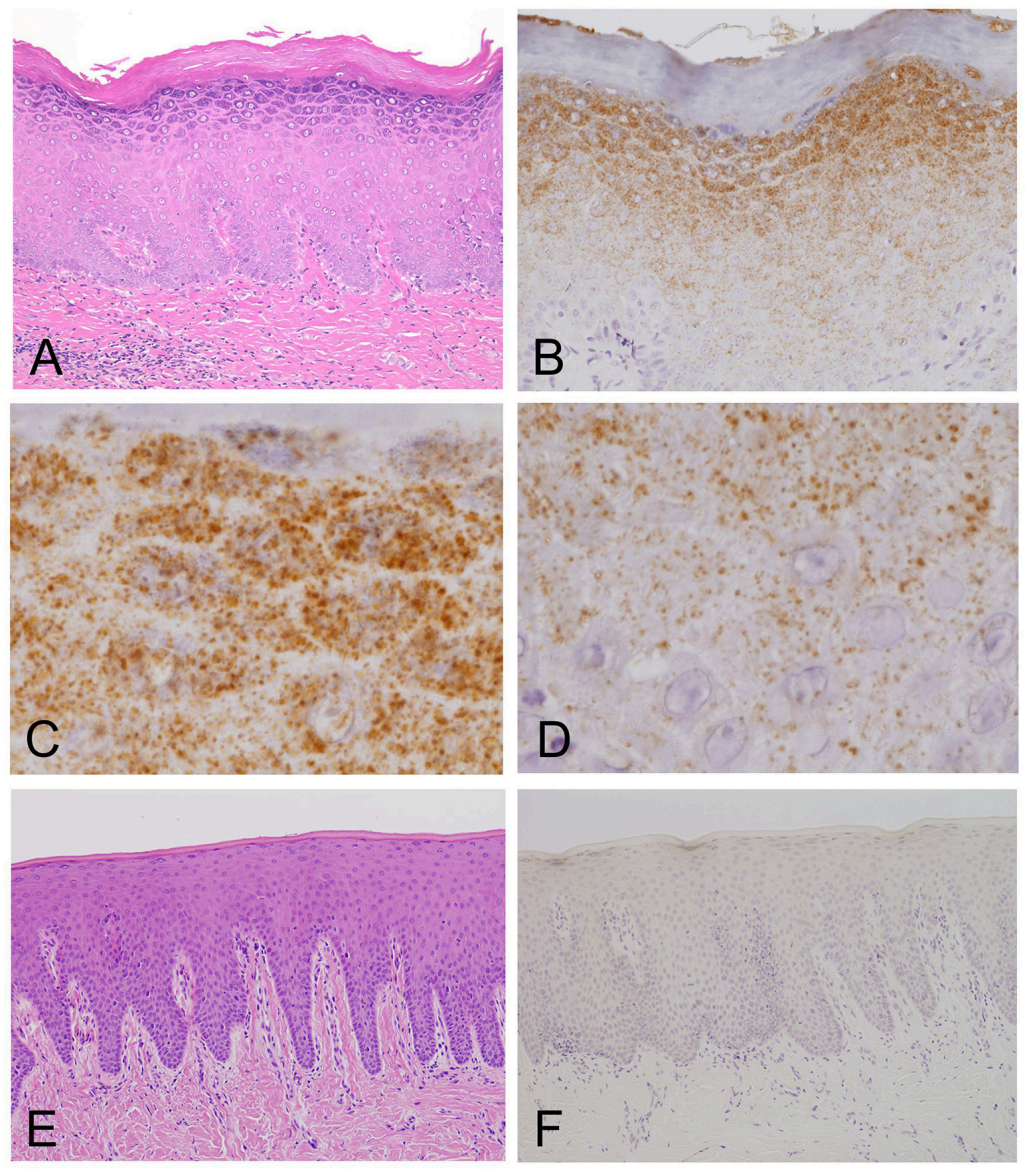
Light microscopy and immunohistochemistry of *Mycoplasma salivarium*. **(A)** A photomicrograph of a hematoxylin–eosin stained tissue section of oral mucosal tissue with oral leukoplakia by light microscopy. The section shows hyperplasia and hyperkeratosis, but there is no dysplasia and Candida is not present (×100). **(B–D)** Immunohistochemistry of an adjacent section to that shown in **(A)**. **(B)** Immunohistochemistry shows that the antibody to *M. salivarium*, which is demonstrated as numerous fine granular stained areas (brown), localizes from the middle part to the upper part of the epithelium, except the keratinized layer (×400). **(C)** Immunohistochemistry showing that the antibody to *M. salivarium* localizes at many sites in the granular layer (×1,000). **(D)** Immunohistochemistry showing the antibody to *M. salivarium* localizes at fewer sites in the middle part of the epithelium than at the upper part of the epithelium (×1,000). **(E)** Hematoxylin and eosin staining for light microscopy of a section of normal appearing oral mucosal without hyperkeratosis and epithelial dysplasia (×100). **(F)** Immunohistochemistry of an adjacent section to that shown in **(E)** shows no positive immunostaining with the antibody to *M. salivarium* in the epithelium (×100).

Light microscopy of the H&E-stained tissue sections of the oral mucosal biopsies showed hyperplasia and hyperkeratosis, but no dysplasia and no colonization with Candida, consistent with oral leukoplakia (Figure 2A).

The results of IHC of the oral leukoplakia tissues using a monoclonal antibody against *Mycoplasma salivarium* showed fine, granular, brown immunostaining in the epithelium. This immunostaining was most intense in the granular layer and decreased with increasing depth from the mucosal surface (Figure 2B). The size of the brown immunostained granules varied, but there was a tendency for those in the upper area to be larger, while those in the lower area were smaller (Figures 2C,D). The keratinized layer showed no immunostaining with antibodies to *M. salivarium* (Figure 2B). IHC of the normal control oral mucosa showed little to no immunostaining in the epithelium (Figures 2E,F).

### Electron microscopy: the standard method

**Figure 3:**
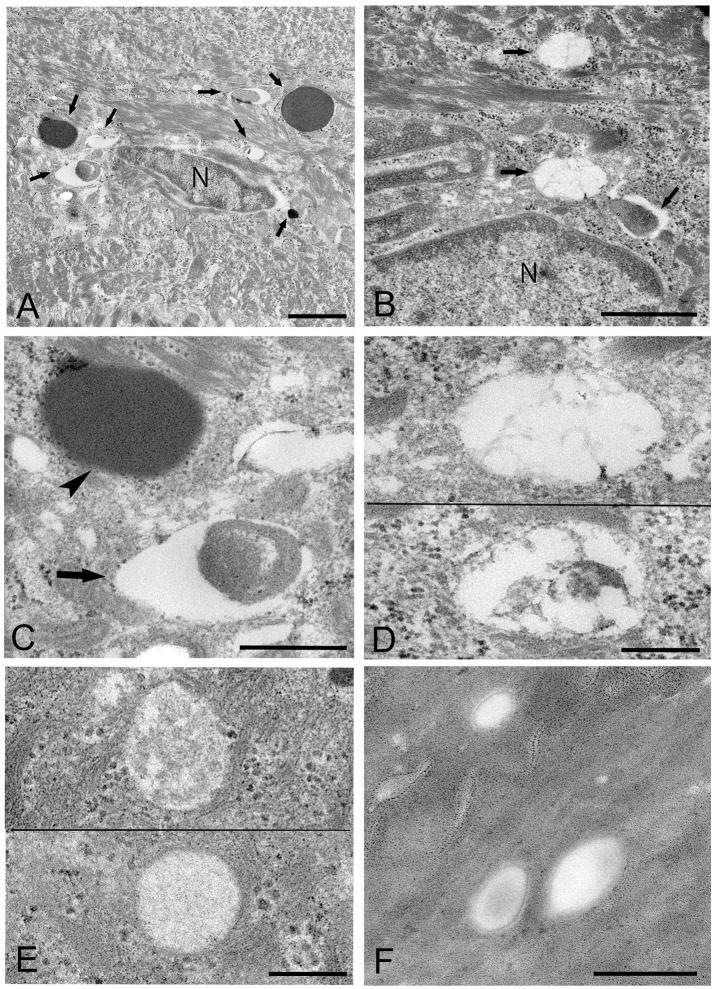
Electron microscopic images of the upper part of the oral mucosal epithelium in samples of oral leukoplakia prepared by the standard electron microscopy method. **(A,B)** Round or oval *Mycoplasma* spp. images with various types of internal structures are observed. Arrows indicate several types of *Mycoplasma* spp. images. Scale bar: 2 μm **(A)**, 1 μm **(B)**, N: nucleus. **(C)** Large *Mycoplasma* spp. images shown in **(A)**. The arrowhead indicates a *Mycoplasma* spp. image containing homogeneous and high electron-dense internal components and the arrow indicates that containing internal components composed of both electron-dense and empty areas. Scale bar: 0.5 μm. **(D)** *Mycoplasma* spp. images containing many vacuoles within an image. Scale bar: 0.2 μm. **(E)** *Mycoplasma* spp. images having low electron-dense internal components with numerous fine granules (lower) or many clusters of fine granules (upper). Scale bar: 0.2 μm. **(F)** Round or ovoid structures appearing to be complete vacuoles in the keratinized layer, which are most likely to be the remains of dead *Mycoplasma* spp. cell. Scale bar: 0.5 μm.

**Figure 4:**
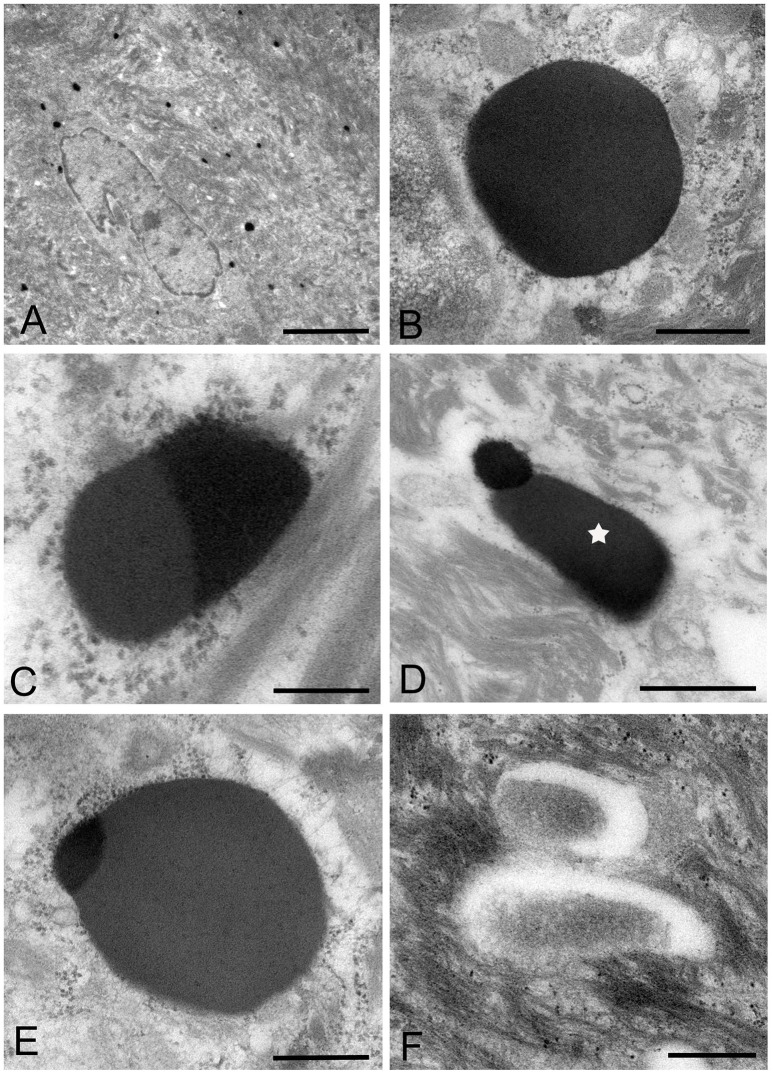
Electron microscopic images of the middle part of the oral mucosal epithelium in samples of oral leukoplakia, which are prepared by the standard electron microscopy method. **(A)** Many *Mycoplasma* spp. cells are observed in the cytoplasm of the epithelial cells as round or ovoid electron-dense structures. Scale bar: 4 μm. **(B)** An image of *Mycoplasma* spp. cell with high electron density, which is morphologically similar to a large electron-dense image shown in Figure 3C. Its border is clear or poorly defined. Scale bar: 0.2 μm. **(C)** An image of *Mycoplasma* spp. cell composed of two parts, one with high electron density and the other with slightly lower electron density, which seems to be in the process of cell division. Scale bar: 0.5 μm. **(D)** An image of an electron-dense structure coupling with a slightly lower electron-dense structure (daughter cell) (asterisk), which seems to demonstrate cell division of *Mycoplasma* spp. Scale bar: 0.5 μm. **(E)** A large, slightly lower electron-dense image including a small area with high electron density in the middle part of the prickle cell layer. This image appears to be a *Mycoplasma* spp. cell expanding from a small, high electron-dense structure to a large structure with slightly low electron density. Scale bar: 0.5 μm. **(F)** An image of twin structures, which seem to be on the process of cell division. Scale bar: 0.5 μm.

*Mycoplasma* spp. were seen as almost round or ovoid images and varied in size from 100 nm or less to 1,000 nm or more in diameter and varied in number between tissue samples and within areas of the epithelium. Based on the ultrastructural morphological internal features, four morphological types of structure were identified: (1) a high electron-dense type containing amorphous and dense internal components (Figures 3A,C, 4A,B); (2) a bubble-like type containing many vacuoles in each cell (Figures 3B,D); (3) a combination-appearance type with internal components composed of both electron-dense area and vacuoles in each cell (Figures 3A–C); and (4) a low electron-dense type containing low electron-dense internal components (Figure 3E). Of the four types of ultrastructural appearance, the bubble-like appearance may be a degenerated form of *M. salivarium*, if the vacuoles are produced by a decrease of ribosomes and/or DNA of the organism near the final stage of its life cycle. Apart from the high electron-dense type, these images were frequently found in the upper part of the stratum spinosum (prickle cell layer) and the stratum granulosum (granular layer) but varied in number according to the sample or the area of a sample.

Many small, round or ovoid images with high electron density were observed in the epithelial cells mainly in the middle part of the prickle cell layer (Figures 4A,B). These images were similar to large images *Mycoplasma* spp. appeared to be replicating within the epidermis, as shown by the presence of: (1) high electron-dense images composed of two parts, one with high electron density and the other with slightly lower electron density (Figure 4C), or (2) high-density images coupling with a slightly lower electron-dense image (Figure 4D). Large, slightly lower electron-dense images, which include a small high electron-dense area at a periphery of the image, were found mainly in the middle part of the prickle cell layer of the epithelium, and it is possible that the small structures with high electron density expanded to form the large structures with slightly lower electron density (Figure 4E). The images, which appeared to be two large cells joined by a narrow area, were found in the upper part of the prickle cell layer and the granular layer (Figure 4F).

In the keratinized layer, many round or ovoid vacuoles were observed, which were most likely to be the remains of dead micro-organisms (Figure 3F), based on their size, round or ovoid shape, empty internal components, and lack of positive reactivity using IHC and IEM. Living or intact *Mycoplasma* spp. were not found in this layer. These findings may indicate that *Mycoplasma* spp. does not invade from the surface into the epithelium through the keratinized layer.

### IEM: the ultracryotomy immunolabeling method

**Figure 5:**
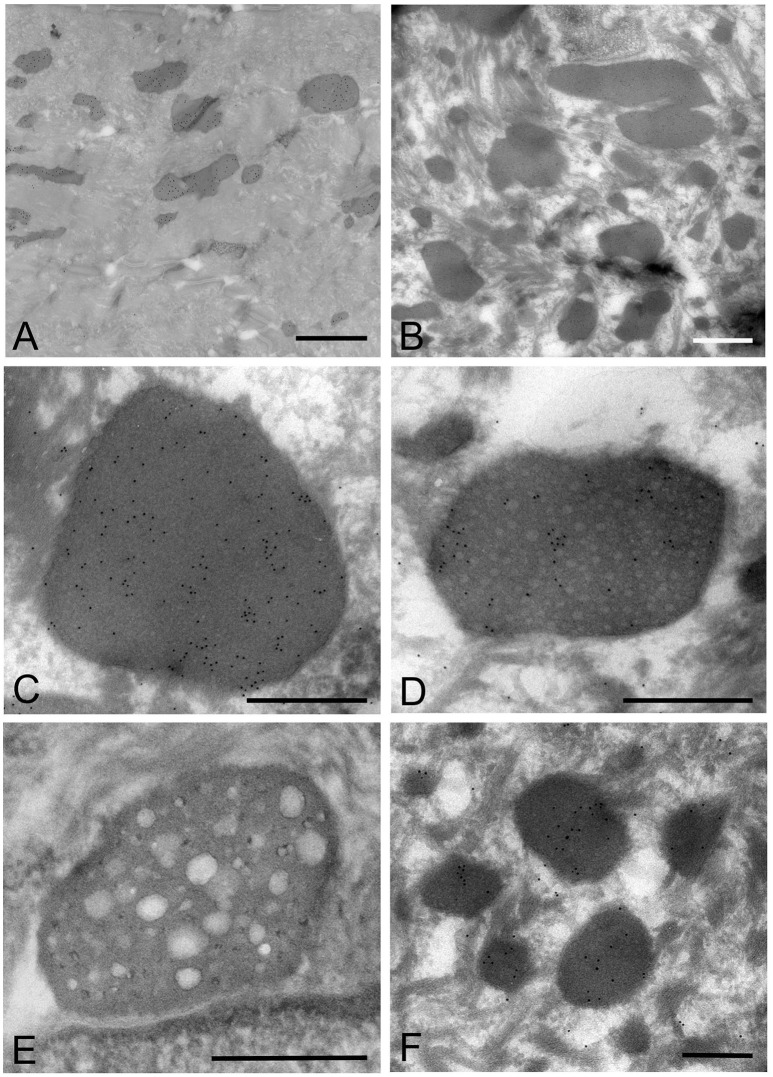
Electron microscopic images of the oral mucosal epithelium in samples of oral leukoplakia prepared by the ultracryotomy-immunolabeling method. **(A,B)** *Mycoplasma* spp. cells are observed as pleomorphic, electron-dense images at low magnification, which vary in size. Scale bar: 2 μm. **(C)** High magnification image of a *Mycoplasma* spp. cell with homogeneous and high electron-dense internal components. Scale bar: 0.5 μm. The border of the image is clear or poorly defined. **(D)** An image of *Mycoplasma* spp. with internal components containing numerous small lower electron-dense granules. Scale bar: 0.5 μm. **(E)** An image of *Mycoplasma* spp. with less electron-dense internal components containing many holes, which vary in number and size. Scale bar: 0.5 μm. **(F)** Small, round or oval images of *Mycoplasma* spp. cells with homogeneous and electron-dense internal components in the middle part of the epithelium. Scale bar: 0.2 μm.

*Mycoplasma* spp. organisms within the cytoplasm of oral mucosal epithelial cells were observed as polymorphous electron-dense images bound by some of gold particles, which were seen in the cells from the middle part of the prickle cell layer to the granular layer of the epithelium. The size of the *M. salivarium* organisms varied from approximately 100 nm to microns in diameter or length, and their electron density was almost uniform at low magnification (Figures 5A,B). Although most of the internal components of the micro-organisms seemed to be homogeneous at low magnification, they varied in appearance and electron density at high magnification (Figures 5C–E).

According to the internal structures identified, the images could be categorized into several types: a type with homogeneous and dense internal components (Figure 5C); a type with internal components containing numerous small low electron-dense granules (Figure 5D); a type containing many holes with various sizes within the cell, rather like a “Swiss cheese” (Figure 5E). Also, small structures with denser internal components than those of large images were observed mainly at the middle part of the epithelium, and their border was clear or poorly defined (Figure 5F).

**Figure 6:**
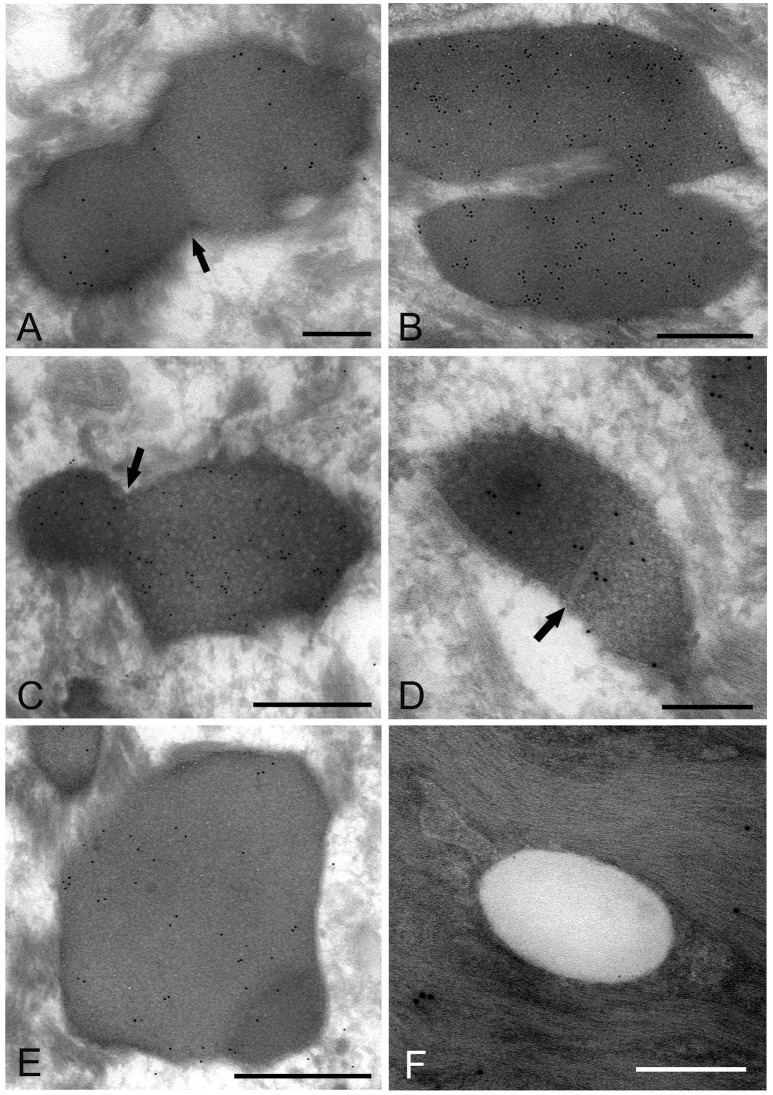
Electron microscopic images of *Mycoplasma* spp. cells, which seem to be in the process of cell division **(A–E)** and localize in the keratinized layer **(F)**, by the ultracryotomy-immunolabeling method. **(A)** An image with the appearance of invagination of the membrane, which seems to be in the process of cell division in binary fission. The arrow indicates invagination of the membrane. Scale bar: 0.5 μm. **(B)** An image of two cells connected by a narrow area, which seems to be on the final stage of cell division. Scale bar: 0.5 μm. **(C)** An image of a cell dividing in binary fission by invagination, which seems that a daughter cell is dividing from an elementary body. Scale bar: 0.5 μm. The arrow indicates invagination of the membrane. **(D)** An image showing formation of a septum across the structure, which is recognized to be in the process of binary fission. The arrow indicates a septum across the structure. Scale bar: 0.2 μm. **(E)** A large and slightly lower electron-dense image including a small area with high electron density in the middle part of the prickle cell layer. This image appears to be a *Mycoplasma* spp. cell enlarging from a small, high electron-dense structure to a large structure with slightly low electron density. Scale bar: 0.5 μm. **(F)** An image of round or ovoid vacuole in the keratinized layer, which is not bound by gold particles. Scale bar: 0.2 μm.

The images showing invagination of the bound membrane (Figures 6A–C), and formation of a cross-septum (Figure 6D), which seemed to show the process of cell division, were found. These appearances were consistent with the known method of replication of *Mycoplasma* spp., by equal or unequal binary fission. In addition, large, slightly lower electron-dense images including a small area with high electron density were also found mainly in the middle part of the prickle cell layer (Figure 6E). These images were similar to those observed by the standard EM method. In the keratinized layer, many round or ovoid vacuoles were observed, which were not bound by gold particles (Figure 6F).

### IEM: the LR white resin embedding and immunolabeling method

**Figure 7:**
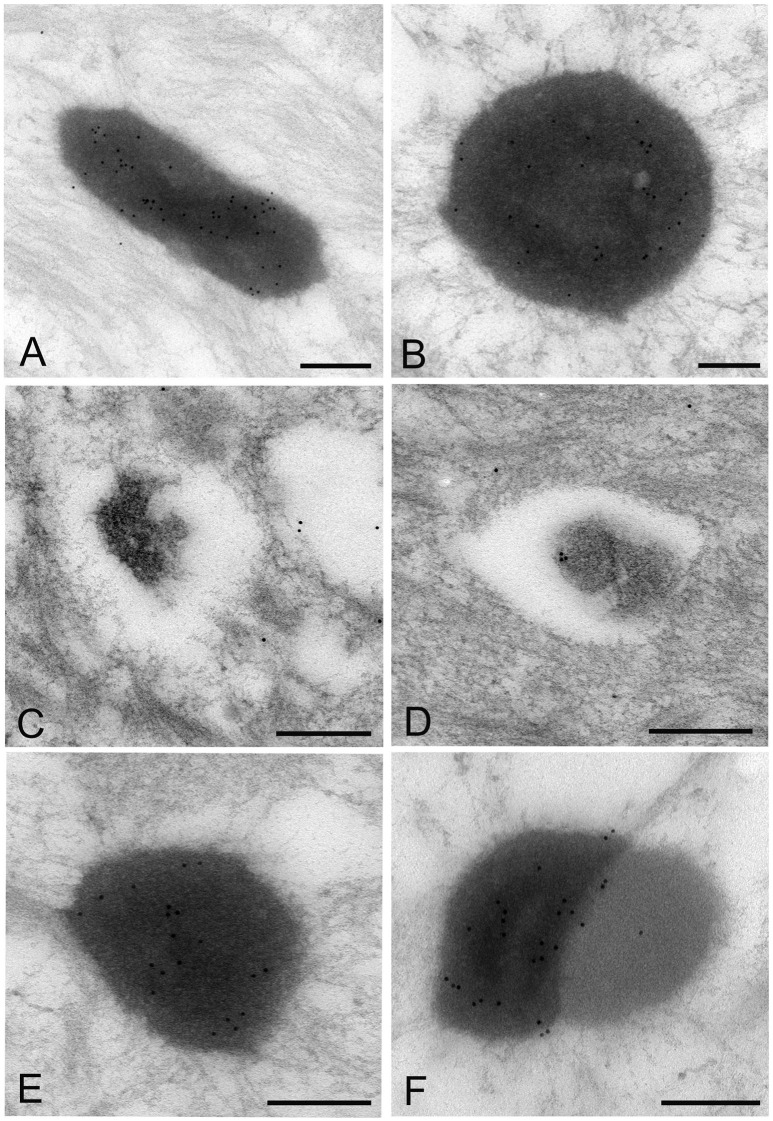
Electron microscopic images of *Mycoplasma* spp. by the LR White resin embedding-immunogold labeling method. These images are very similar to those identified by the standard electron microscopy method. Images of *Mycoplasma* spp. are bound to gold particles, with a variable amount of the image or part of an image. Many particles are attached on the electron-dense images or areas, while fewer or no particles are attached to the low electron-dense images or areas and vacant areas. Scale bar: 0.5 μm **(A,B)**, 0.2 μm **(C–F)**.

The images of *Mycoplasma* spp. detected by the LR White resin embedding-immunolabeling method were very similar to those described using the standard method (Figures 7A–F). Although the number of gold particles varied among the micro-organisms, or part of the micro-organism, images of *Mycoplasma* spp. were identified as being bound to the gold particles (Figures 7A–F).

High electron-dense images observed by the standard method were confirmed to be *Mycoplasma* spp. by this method. The borders of the images of the Mycoplasma were less well-defined than those of images produced by the standard method. Further, transformation of mycoplasma cells was less than that by the standard method. This may result from the difference of fixation method and embedding material.

## Discussion

This study was undertaken to clarify and detail the ultrastructure of the life cycle of *M. salivarium* in tissue biopsies of oral leukoplakia by three different methods. Although the EM images of *M. salivarium* in the epithelial cells of oral leukoplakia tissue depended on the method, four ultrastructural appearances of *M. salivarium* were obtained: (1) small, electron-dense cellular-like structures, or elementary bodies of *M. salivarium*; (2) large structures of *M. salivarium*; (3) *M. salivarium* organisms in cell division; (4) the sequence of events in the life cycle of *M. salivarium* in the epithelium of oral leukoplakia, including: (a) elementary bodies of *M. salivarium* deep in the oral mucosal epithelium; (b) replication by binary fission and daughter cell division from the elementary bodies; (c) maturation or degeneration of *M. salivarium* in the epithelial cells mainly at the upper part of the epithelium; and, (d) death of the organisms in the granular and/or keratinized layer.

**Figure 8:**
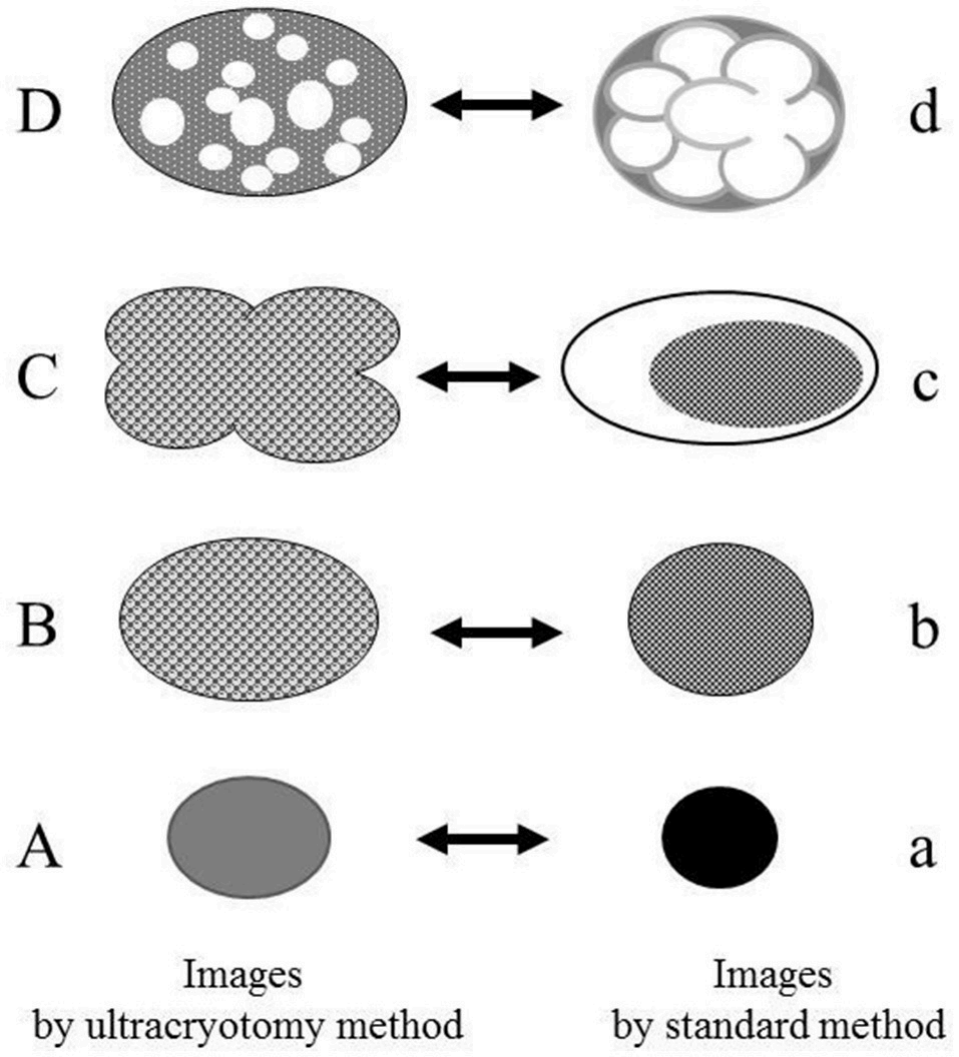
The relationship between the electron microscopic images of the *Mycoplasma* spp. by the ultracryotomy method and those by the standard electron microscopy method. The differences between images resulting from these techniques are considered to result from the discrepancy of the degree in shrinking and transformation of the micro-organisms, which was caused by fixation, dehydration, and embedding. High electron-dense images **(a)** are produced from the cells with amorphous and dense internal components **(A)**. Low electron-dense images **(b)** are produced from the cells with less dense internal components **(B)**. Combination images **(c)** are assumed to be produced from pleomorphic cells **(C)**. The ratio of the cell surface area and volume of the internal component is larger for the pleomorphic cells **(C)** than the spherical or oval cells **(B)**. That is, when the volume of the internal component is the same between the cell **(B)** and the cell **(C)**, the surface area of the cell **(C)** is larger than the cell **(B)**. Therefore, the discrepancy between the capacity of a cell and the volume of internal components may have arisen by shrinking and transformation from a pleomorphic to a spherical or oval shape. Vacuolated images **(d)** may be produced from the *Mycoplasma* spp. **(D)**, which contain many vacuoles within a cell (like a “Swiss cheese”), by shrinking of internal components among the vacuoles.

The images produced by the standard method, were similar to those by the LR White resin embedding method, but were very different from those obtained by the ultracryotomy method. The differences in the images obtained by these three methods may be due to the differences in fixation, dehydration and embedding that may cause shrinking and transformation of the *M. salivarium* (Woldringh, 1973; Loqman et al., 2010; Shah et al., 2015; Figure 8). Since the ultracryotomy method does not include the dehydration and resin embedding, it may preserve the natural shape and internal structure of *M. salivarium* better than the other two methods. In contrast, the degree of shrinking and transformation of *M. salivarium* using the standard method may be the most pronounced among three methods due to fixation with paraformaldehyde/glutaraldehyde and osmium tetroxide, and embedding in epoxy resin (Shah et al., 2015). The LR White resin embedding method was useful for IEM to identify *M. salivarium* in the epithelial cells of oral leukoplakia tissue; this method for IEM is easier to perform than the ultracryotomy-immunolabeling method.

The EM images, which were obtained in this study, provided a useful reference for ultrastructural identification of *M. salivarium* in cells and tissues. Previous studies showing ultrastructural images of oral leukoplakia using EM have described the presence of structures that include keratohyalin granules (keratin aggregates), seen as electron-dense granules, lipid bodies (lipid granules or lipid droplets), seen as vacuoles, or Odland bodies, seen as membrane-bound organelles (Silverman, 1967; Hashimoto et al., 1968; Banoczy et al., 1980; Rodriguez et al., 1984; Jungell et al., 1987; Kannan et al., 1993; Tamgadge et al., 2012). Although these previous studies have not reported the presence of micro-organisms, including Mycoplasma, we propose that these previously described structures may represent phases in the life cycle of *M. salivarium* in the epithelial cells of oral leukoplakia. That is, keratohyalin granules, lipid bodies, and Odland bodies may represent high electron-dense images, bubble-like images, and combination-appearance images or low electron-dense images of *M. salivarium*, respectively.

Previous studies that have described the ultrastructural appearances of Mycoplasma organisms (grown in broth or on agar) by EM using the standard method have shown the presence of very small electron-dense granules or bodies, which were previously referred to as “elementary bodies” or “minimal reproductive units” (Domermuth et al., 1964; Anderson and Barile, 1965; Hummeler et al., 1965; Knudson and MacLeod, 1970; Nakamura and Kawaguchi, 1972; Furness et al., 1976). In this study, the high electron-dense images corresponding to elementary bodies were observed by each EM method used, seen as structures with homogeneous and electron-dense internal components by the ultracryotomy method but with a density that was not as high as that produced by other methods. High electron density of these structures, which were seen by the standard method and the LR White resin embedding method, may be produced by high contraction of the internal components of *M. salivarium*, which is caused by fixation, dehydration or embedding in the resin.

The presence of elementary bodies suggests that replication of *Mycoplasma* occurred. The replication of *M. salivarium* was confirmed by the images combining a high electron-dense part attached to a low electron-dense part, or “daughter cells” observed using the standard method of EM. Several reproductive modes for *M. salivarium* are reported, including binary fission, the fragmentation of elongated organisms, and budding (Miyata, 2002). The reproductive mode of *M. salivarium* in the epithelial cells of oral leukoplakia tissue was considered to be the same as that reported when the organism is grown in broth or agar, that of binary fission (Furness, 1970). The replication mode of *M. salivarium* in the epithelial cells was confirmed to be by equal or unequal binary fission by images showing invagination of membranes and formation of a cross-septum, when using the ultracryotomy immunolabeling method. A previous study that investigated the cell division of T-Mycoplasma by EM reported that invagination of bound membrane was seen, but no cross-septum was seen (Whitescarver and Furness, 1975). In this study, both invagination and cross-septum formation were observed. The fragmentation of elongated cells or budding from the large mature *M. salivarium* was not found. Also, the division of large *M. salivarium* by binary fission was also observed in the upper part of the prickle cell layer and the granular layer. These observations may explain the findings that immunostaining was most intense in the granular cell layer and decreased with increasing depth from the mucosal surface. We believe that this study is the first to demonstrate the presence of *M. salivarium* elementary bodies and that the replication mode of *M. salivarium* in the epithelial cells of oral leukoplakia tissue is binary fission.

**Figure 9:**
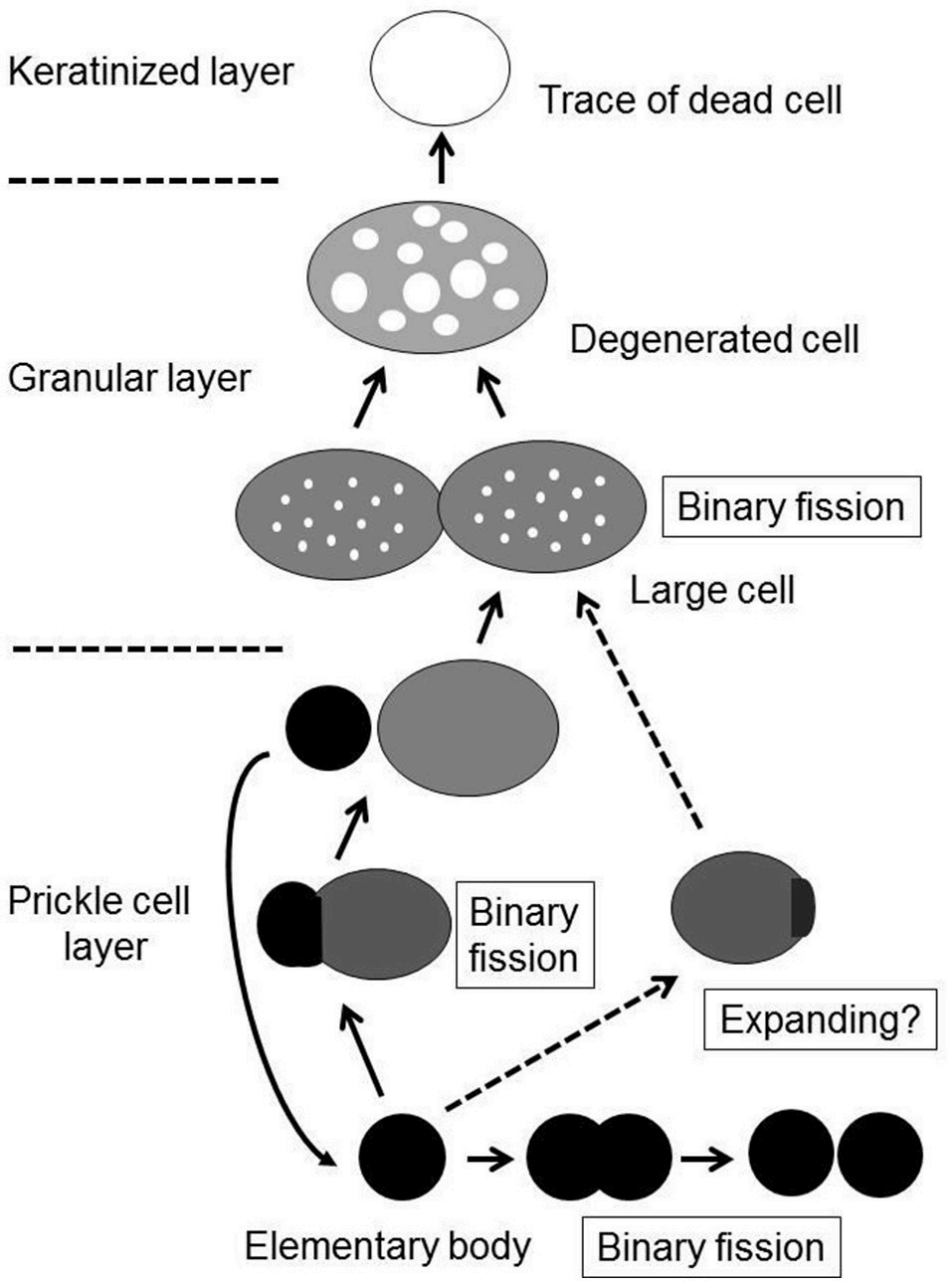
The proposed life cycle of *Mycoplasma salivarium* in the epithelial cells of the oral mucosa in oral leukoplakia. The elementary bodies of *Mycoplasma salivarium* invade into the surface epithelium of the oral mucosa without hyperplasia/hyperkeratosis, and through the epithelial cells and/or via the intercellular spaces, to replicate in the epithelial cells mainly at the middle part of the epithelium. Daughter cells divide from the elementary bodies by binary fission, mature to large cells and degenerate. The maturation and degeneration may proceed in the epithelial cells with migration from the middle part to the upper part of the epithelium, where they die in the granular layer and/or keratinized layer. Maturation of elementary bodies to large cells by expanding is also suggested. This proposed sequence of events in the life cycle of *Mycoplasma salivarium* in the epithelial cells of the oral mucosa in oral leukoplakia is derived from the observations made in the present study.

Although the process of epithelial colonization by *M. salivarium* and its life cycle in the oral mucosa is still not completely understood, and requires further investigation, we can deduce the following sequence of events from the findings of this study. The elementary bodies of *M. salivarium* invade into the surface epithelium of the oral mucosa without hyperkeratosis, and through the epithelial cells and/or via the intercellular spaces, to replicate in the epithelial cells mainly at the middle or lower part of the epithelium. Daughter cells divided from the elementary bodies by binary fission, to become large cells that multiply by binary fission, mature and degenerate in the epithelial cells with migration from the middle part to the upper part of the epithelium, where they die in the granular layer and/or keratinized layer (Figure 9). This proposed life cycle of *M. salivarium* in the oral mucosal epithelium remains to be supported by further studies.

This was a small study using five oral leukoplakia samples and was limited to the morphological observation of *M. salivarium*. Therefore, the relationship between infection with *M. salivarium* of the epithelium of the oral mucosa and the development of oral leukoplakia could be explained by these ultrastructural images of the life cycle of *M. salivarium*. Further, larger studies are recommended to examine the possible role of *M. salivarium* in the pathogenesis of oral leukoplakia.

In conclusion, several types of electron microscopic images of *M. salivarium* in the epithelial cells of oral leukoplakia tissue, which include images of the elementary body and cell division of *M. salivarium*, were demonstrated in this study. Based on those images, we have described the possible sequence of events in the life cycle of *M. salivarium* in the epithelial cells of oral leukoplakia tissue. These ultrastructural images may provide a useful reference for the identification of *M. salivarium* in diagnostic cytology or biopsy material.

## Author contributions

HM designed the study and wrote the initial draft of the manuscript. HM and RA contributed to acquisition of materials. HM, RA, and TM interpreted the data. RA and TM performed the literature review.

### Conflict of interest statement

The authors declare that the research was conducted in the absence of any commercial or financial relationships that could be construed as a potential conflict of interest.
